# Macrophage Gal/GalNAc lectin 2 (MGL2)^+^ peritoneal antigen presenting cells during *Fasciola hepatica* infection are essential for regulatory T cell induction

**DOI:** 10.1038/s41598-022-21520-w

**Published:** 2022-10-21

**Authors:** Monique Costa, Valeria da Costa, Pablo Lores, Mercedes Landeira, Santiago A. Rodríguez-Zraquia, María Florencia Festari, Teresa Freire

**Affiliations:** Laboratorio de Inmunomodulación y Vacunas, Departamento de Inmunobiología, Facultad de Medicina, Universidad de La República, Gral. Flores 2125, 11800 Montevideo, Uruguay

**Keywords:** Immunology, Infectious diseases, Parasitic infection

## Abstract

*Fasciola hepatica*, one of the agents that causes fasciolosis, modulates the host immune system to allow parasite survival in the host. *F. hepatica* expresses carbohydrate-containing glycoconjugates that are decoded by C-type lectin receptors, such as Dectin-1, mannose receptor, DC-SIGN and MGL, that are mainly present on myeloid antigen presenting cells (APCs) and can mediate immunoregulatory properties on T cells. In particular, Macrophage Gal/GalNAc lectin 2 (MGL2) expands modified Th2 immune responses, while suppressing Th1 polarization, upon recognition of GalNAc-glycosylated parasite components. In this study, by using MGL2-DTR transgenic mice that encode human diphtheria toxin receptor in MGL2^+^ cells, we demonstrate the role of peritoneal APCs during *F. hepatica* infection in favoring parasite survival. This process might be mediated by the induction of splenic Tregs in vivo, since the depletion of MGL2^+^ cells conferred mice with partial resistance to the infection and abrogated the increase of CD4^+^/CD25^+ ^FoxP3^+^ Tregs induced by the parasite. Therefore, MGL2^+^ cells are critical determinants of *F. hepatica* infection and could constitute immune checkpoints to control parasite infection.

## Introduction

*Fasciola hepatica* is a trematode parasite that causes fasciolosis, a zoonotic disease that affects humans^1,2^. It also infects livestock, causing significant economic losses worldwide^1,2^. To survive in its mammalian hosts, *F. hepatica* is capable of modulating the host immune system by inducing a modified type-2 responses characterized by potent immune regulatory processes such as differentiation of regulatory T cells (Tregs), alternative activation of macrophages, involvement of regulatory dendritic cells (DCs), upregulation of IL-10 and TGFβ and down-regulation of Th1 cytokines^3–7^.

Helminths express carbohydrate-containing glycoconjugates that are extremely important in their life cycles and pathogeny since they can participate in immune escape^8^. Indeed, glycoconjugates produced by *F. hepatica* are able to modulate the maturation and function of DCs^9,10^ and macrophages^11–13^. Furthermore, *F. hepatica* glycans participate in parasite migration through the intestine in early stages of the infection^14,15^.

The immunomodulatory role of parasite glycans relies on the ability of lectins to decode their information, such as C-type lectin receptors (CLRs), that are mainly present on myeloid antigen presenting cells (APC)^16,17^. Previously data have demonstrated that CLRs are key mediators of the immunoregulatory properties induced by *F. hepatica*. In fact, Dectin-1 on macrophages interacts with *F. hepatica* excretory-secretory products inducing an alternative activated macrophage phenotype^11,12^ and likely exerting T cell anergy via selective up-regulation of PD-L2 expression on macrophages in a Dectin-1 dependent way^18^. However, in-depth studies on the function of these cells are still necessary^12^. Injection of *F. hepatica* tegumental antigens induces anergic-like T cells via DCs in a mannose receptor (MR)-dependent manner, although the role of MR during *F. hepatica* infection has not been determined so far^19^. In contrast, no implication of the MR was found in the suppression of LPS-induced cytokines by bone marrow derived DCs treated with *F. hepatica* derived-molecules^13^. On the other hand, dendritic cell-specific ICAM-3 grabbing non-integrin (DC-SIGN) interacts with *F. hepatica* glycoconjugates through mannose and fucose residues on regulatory DCs and decreases allogeneic T cell proliferation, via the induction of anergic T cells^10^.

Macrophage Gal/GalNAc lectin 2 (MGL2, CD301) binds to terminal GalNAc residues, including the Tn antigen (GalNAc-αThr/Ser) and is mainly expressed on immature, tolerogenic or type-2 DCs^6,20,21^ and alternatively-activated macrophages^22^. Moreover, MGL activation dampens immune responses, by inducing synthesis of IL-10 by DCs^6,23^, promoting the differentiation of Tregs^24^, inducing T cell apoptosis and suppressing T cell activation^25^. Previous studies from our group demonstrated that human MGL can interact with *F. hepatica* components through the Tn antigen and modulate the TLR2-induced maturation of human monocyte derived DCs by up-regulating the production of IL-10 and TNFα^6^. In addition, we have shown that MGL2^+^ cells in *F. hepatica* infected mice express a variety of regulatory markers, including IL-10, TNFα and TGFβ, expand modified Th2 immune responses and suppress Th1 polarization^6^. However, the role of MGL2^+^ cells during *F. hepatica* infection in inducing Tregs in vivo has not yet been investigated.

In this study, we demonstrate that MGL2-expressing APCs recruited to the peritoneal cavity during *F. hepatica* experimental infection in mice are essential for parasite survival as well as for the induction of splenic Tregs in vivo. We used MGL2-DTR transgenic mice that encode human diphtheria toxin receptor (DTR) in MGL2^+^ cells. Thus, these cells can be depleted with diphtheria toxin (DT) injection^24^. The depletion of MGL2^+^ cells conferred mice with partial resistance to the infection and abrogated the increase of CD4^+^/CD25^+ ^FoxP3^+^ Tregs induced by the infection. Therefore, MGL2^+^ cells could constitute immune checkpoints to control *F. hepatica* infection.

## Results

### *F. hepatica* infection in MGL2-DTR transgenic mice is characterized by the recruitment of immunoregulatory F4/80^+^ cells in the peritoneum and higher levels of splenic Treg

**Figure 1:**
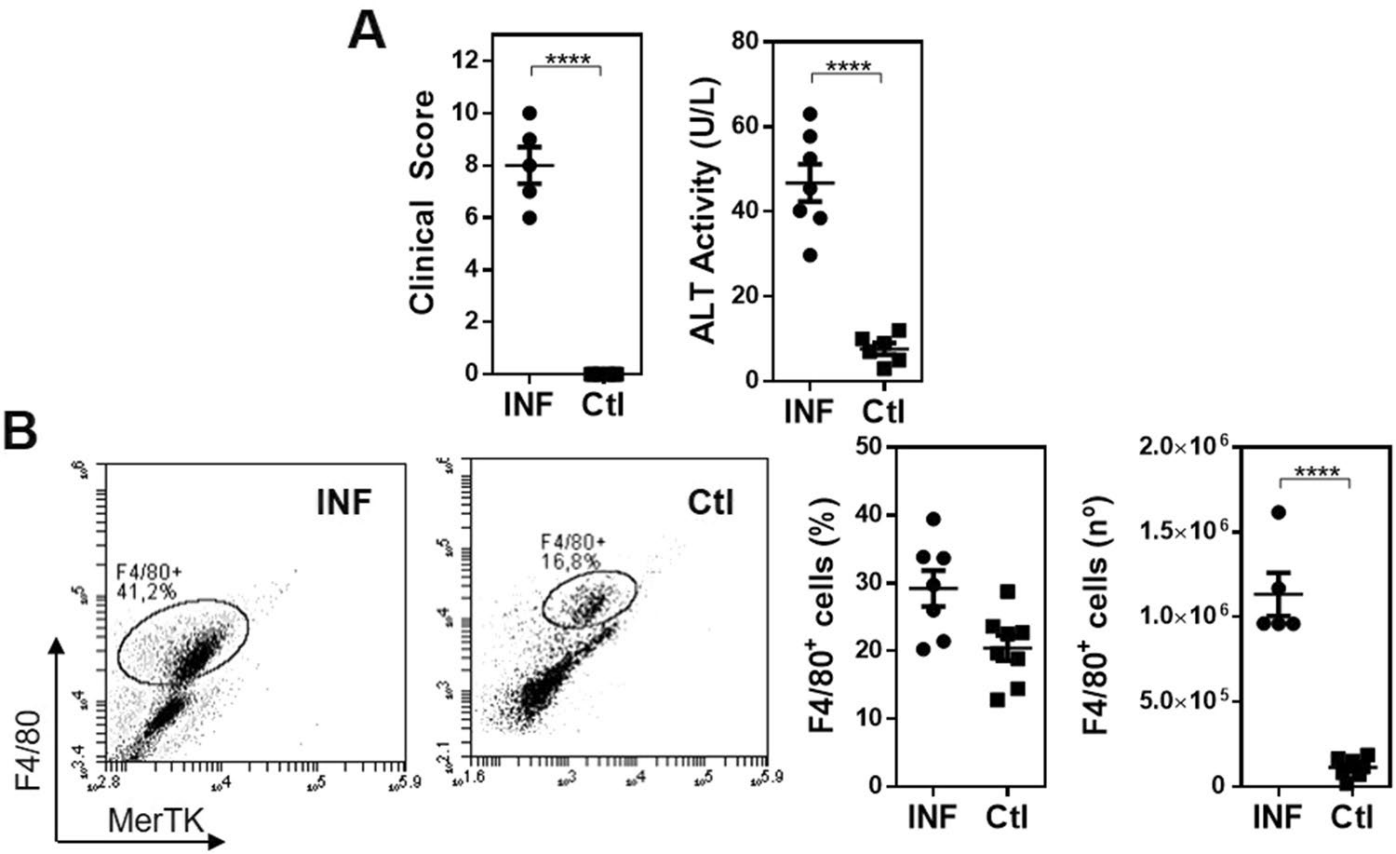
*F. hepatica* infected MGL2-DTR mice present an increase of F4/80^+^ cells in the peritoneal cavity. (**A**) Clinical score and ALT in serum after 3 wpi. MGL2-DTR mice were infected with 10 *F. hepatica* metacercariae (INF). Non infected mice injected with PBS were used as controls (Ctl). (**B**) Frequency and number of F4/80^+^ cells in the peritoneal cavity determined by flow cytometry. A representative figure of three independent experiments is shown (± SEM, indicated by error bars). Asterisks indicate statistically significant differences (****p < 0.0001).

**Figure 2:**
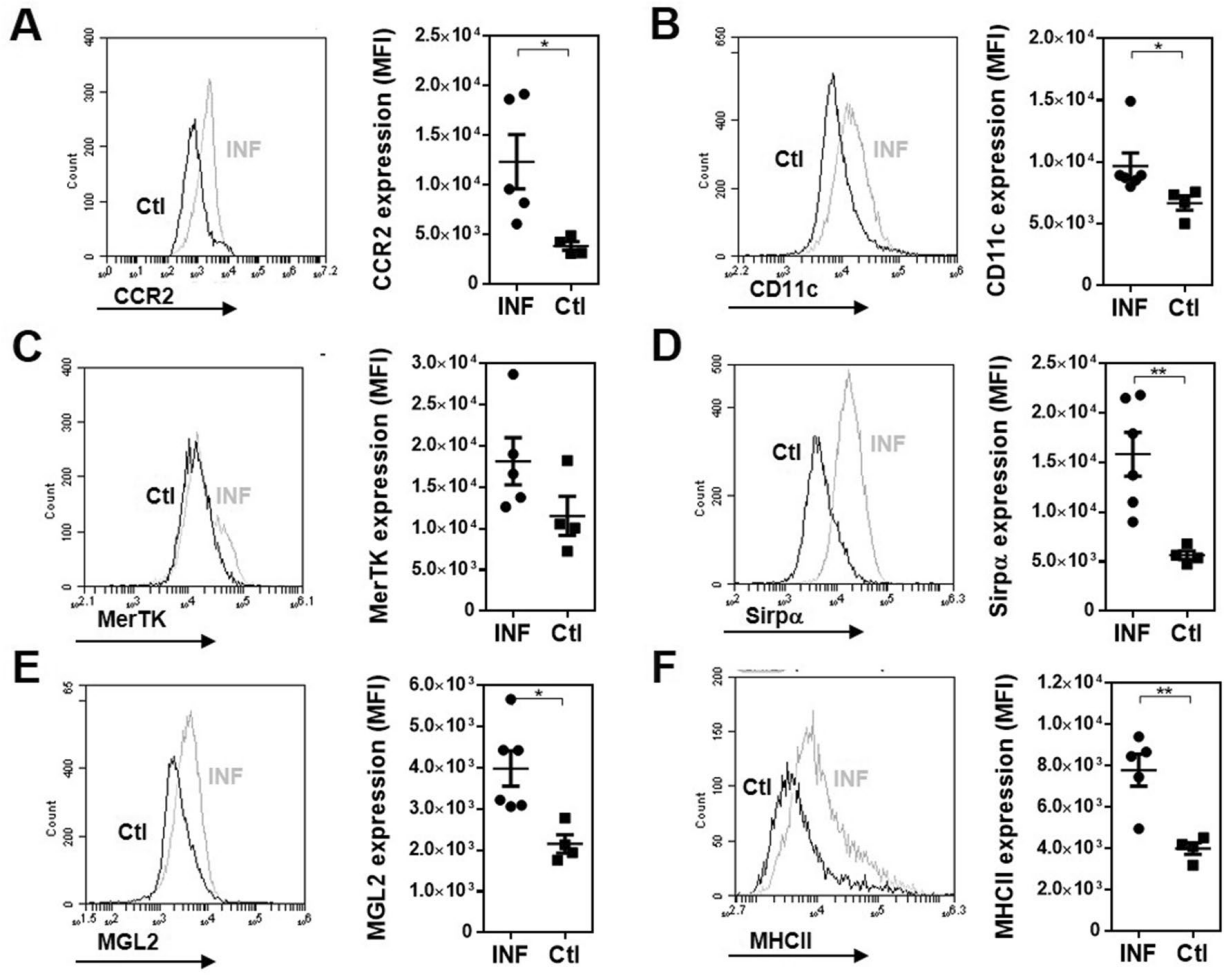
Peritoneal F4/80^+^ cell phenotype in *F. hepatica* MGL2-DTR mice. Expression of CCR2 (**A**), CD11c (**B**), MerTK (**C**), Sirpα (**D**), MGL2 (**E**) and MHC class II (**F**) is shown in F4/80^+^ peritoneal cells from *F. hepatica* infected mice (INF) and non-infected animals (Ctl). Grey lines in histograms represent infected mice while black lines indicated control mice. A representative figure of three independent experiments is shown (± SEM, indicated by error bars). Asterisks indicate statistically significant differences (*p < 0.05, **p < 0.01).

**Figure 3:**
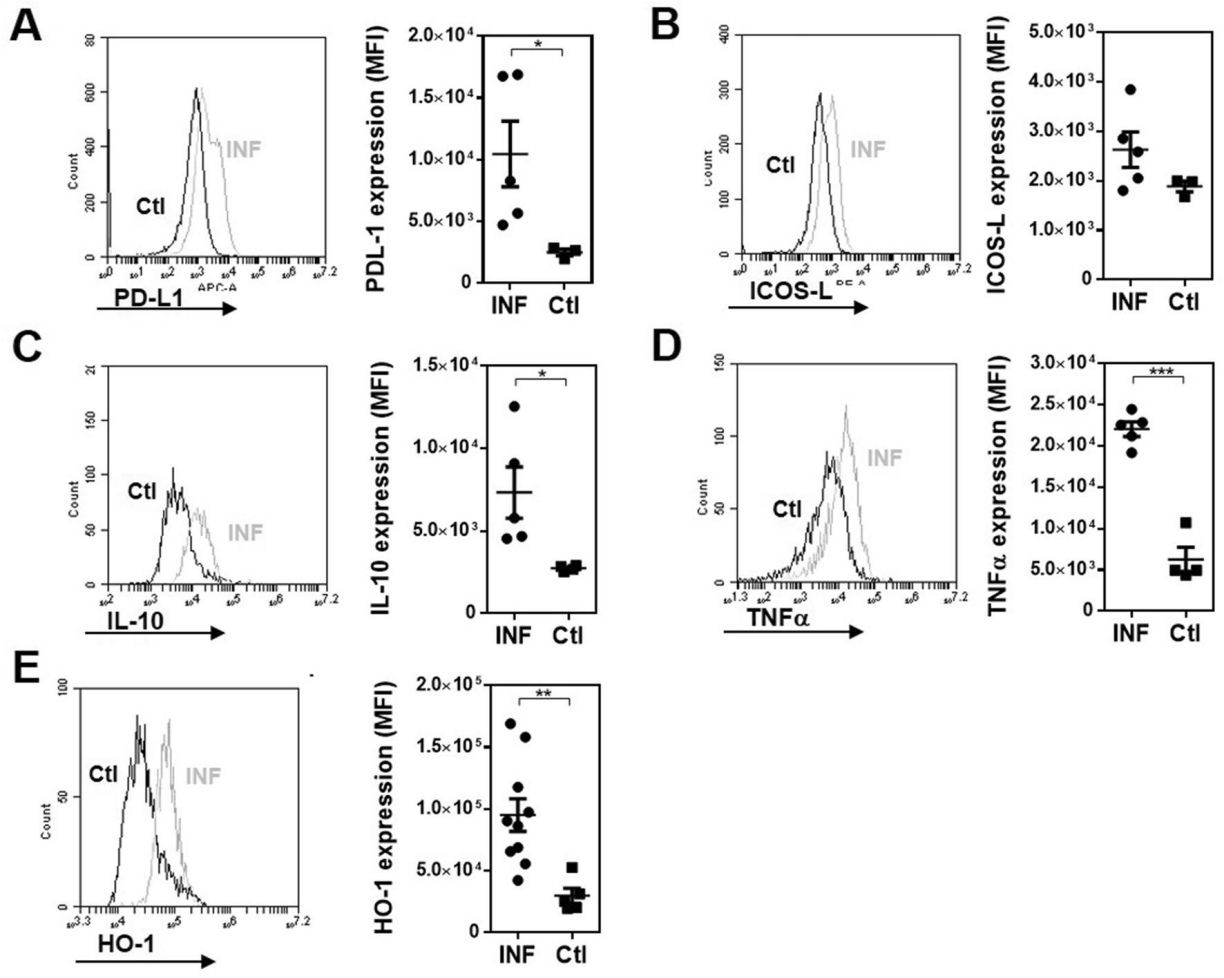
Peritoneal F4/80^+^ cells in *F. hepatica* MGL2-DTR mice express immunoregulatory molecules. Expression of PD-L1 (**A**), ICOS-L (**B**), IL-10 (**C**), TNFα (**D**) and HO-1 (**E**) is shown in F4/80^+^ peritoneal cells from *F. hepatica* infected mice (INF) and non-infected animals (Ctl). Grey lines in histograms represent infected mice while black lines indicated control mice. A representative figure of three independent experiments is shown (± SEM, indicated by error bars). Asterisks indicate statistically significant differences (*p < 0.05, **p < 0.01).

First, we infected MGL2-DTR transgenic mice and analyzed the clinical signs and immunological response induced by *F. hepatica* infection. After 3 weeks post-infection (wpi), infected animals presented a high clinical score, determined by the general state of the animal (Supplementary Table 1) and alanine transaminase (ALT) activity in serum, a common marker to detect hepatic dysfunction, and liver damage and fibrosis (Fig. 1A). Furthermore, infection was associated with an increase of F4/80^+^ cells in the peritoneal cavity of infected mice. However, the frequency of these cells was not significantly different from those determined in non-infected mice (Fig. 1B and Supplementary Fig. 1A). In addition, peritoneal F4/80^+^ cells from infected mice expressed higher CCR2 (Fig. 2A and Supplementary Fig. 1B) and CD11c (Fig. 2B) levels. However, they did not upregulate MerTK expression (Fig. 2C). Additional phenotyping by flow cytometry showed a significantly enhanced expression of Sirpα (Fig. 2D) and MGL2 (Fig. 2E) in infected mice. Furthermore, F4/80^+^ MGL2^+^ cells upregulated MHC class II expression with the infection (Fig. 2F). On the other hand, F4/80^+^ MGL2^+^ peritoneal APCs upregulated different immunoregulatory molecules, such as PD-L1 (Fig. 3A), but not ICOS-L (Fig. 3B). Last, they also expressed higher levels of immunomodulatory molecules such as IL-10 (Fig. 3C), TNFα (Fig. 3D) and hemoxygenase-1 (HO-1) (Fig. 3E).

**Figure 4:**
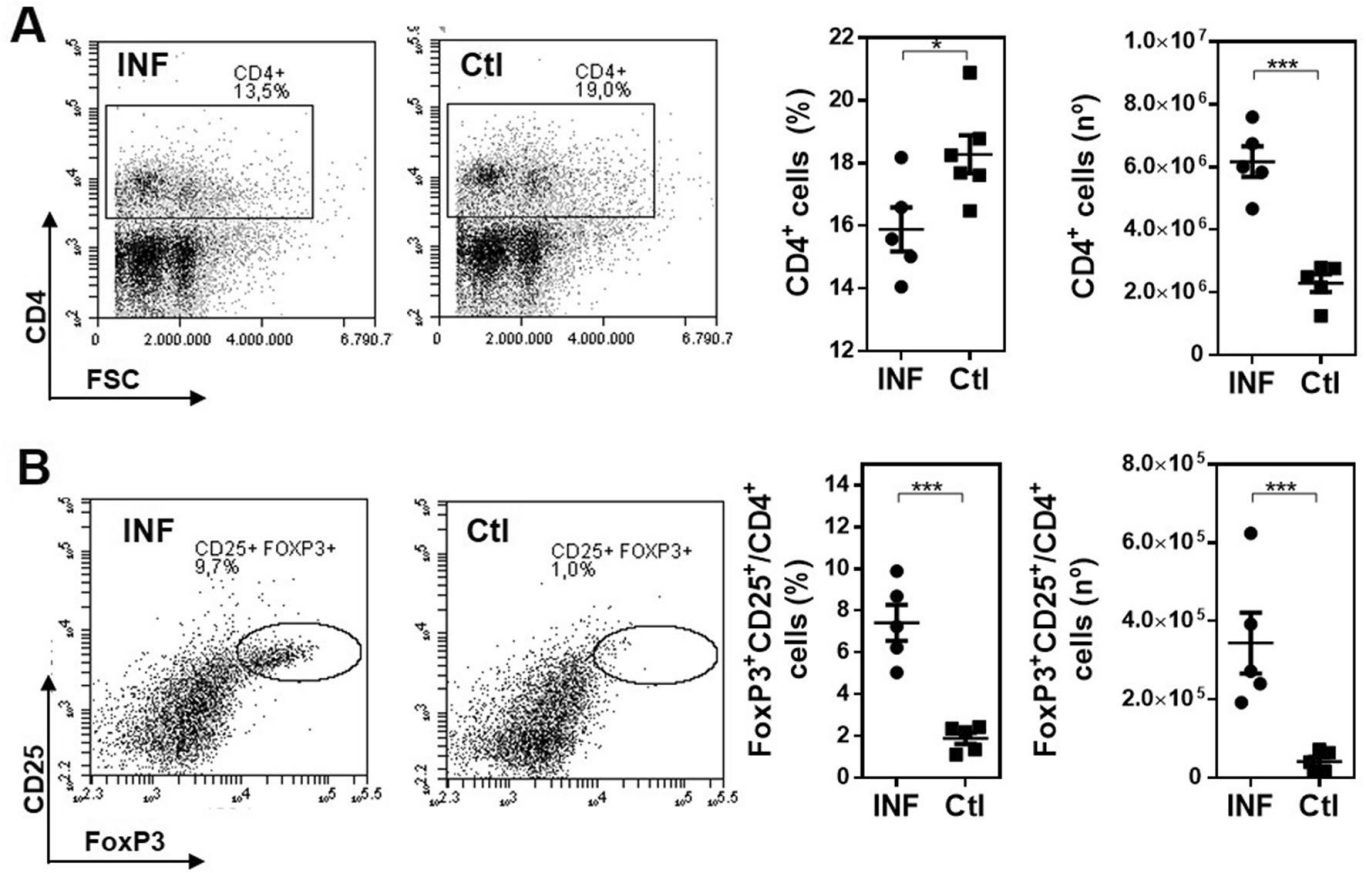
*F. hepatica* infection is associated with an increase in splenic Tregs. Frequency and number of CD4^+^ cells (**A**) and FoxP3^+ ^CD25^+^/CD4^+^ cells. (**B**) in spleens were determined by flow cytometry. Infected MGL2-DTR mice were infected with 10 *F. hepatica* metacercariae (INF). Non infected mice were used as controls (Ctl). A representative figure of three independent experiments is shown (± SEM, indicated by error bars). Asterisks indicate statistically significant differences (*p < 0.05, ***p < 0.001).

Then, we analyzed the presence of Tregs in spleens of 3 wpi MGL2-DTR mice. As depicted in Fig. 4A, a lower frequency of CD4^+^ T cells in spleens of infected mice was detected, although their number was higher than in non-infected mice, likely due to prominent splenomegaly in response to the infection (Supplementary Fig. 2). In addition, FoxP3^+^ CD25^+^ in CD4^+^ T cells increased with infection, both in frequency and number, in relation to non-infected mice (Fig. 4B).

### MGL2^+^ cell depletion is associated with resistance to *F. hepatica* infection

**Figure 5:**
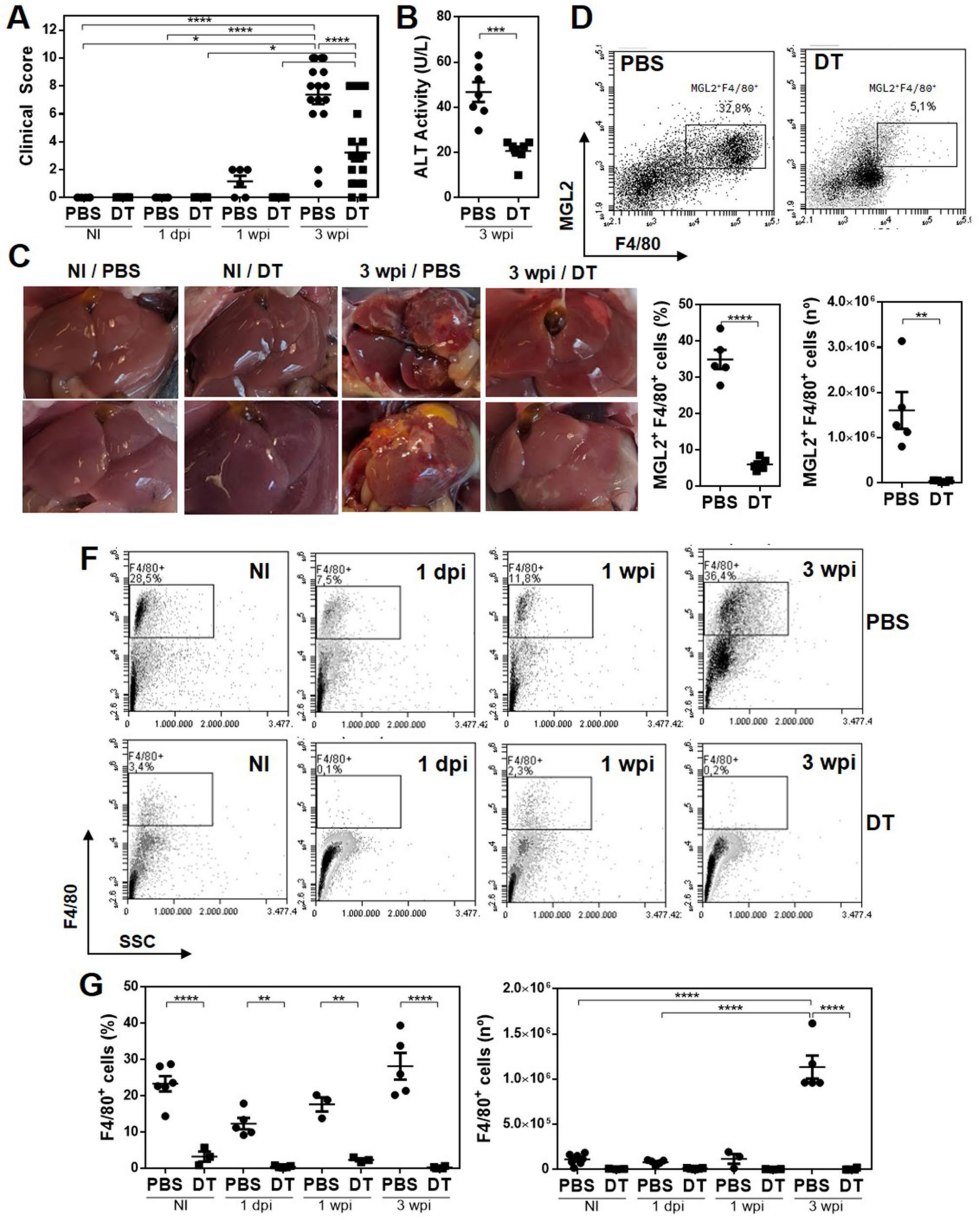
Depletion of F4/80^+^ MGL2^+^ cells during *F. hepatica* infection attenuates the clinical signs induced by the parasite. MGL2-DTR mice were infected with 10 *F. hepatica* metacercariae and sacrificed at 1 dpi, 1 wpi and 3 wpi. MGL2^+^ cells were depleted with DT-treatment. Control mice were injected with PBS. Non infected mice were used as controls (NI). Clinical score (**A**) and ALT (**B**) in serum of infected and control DT- or PBS-treated mice. (**C**) Representative images of livers from DT- or PBS-treated 3 wpi mice. (**D**) Peritoneal F4/80^+^ MGL2^+^ cells gating in *F. hepatica* infected mice treated with DT. (**E**) Frequency and number of MGL2^+^ F4/80^+^ peritoneal cells in infected DT- or PBS-treated mice. (**F**) Peritoneal F4/80^+^ cells gating during *F. hepatica* infection in DT-treated mice. (**G**) Frequency and number of F4/80^+^ peritoneal cells in infected DT- or PBS-treated mice at 1 dpi, 1 wpi and 3 wpi. Data from three independent experiments is shown (± SEM, indicated by error bars). Asterisks indicate statistically significant differences (*p < 0.05, **p < 0.01, ***p < 0.001, ****p < 0.0001).

In order to determine the role of MGL2^+^ F4/80^+^ cells during *F. hepatica* infection, we injected MGL2-DTR transgenic mice with DT one day before and after the infection and every 3 days. Mice depleted of MGL2^+^ cells showed significantly lower clinical signs than control (PBS) mice at 3 wpi (Fig. 5A), which was also related to a significant decrease in serum ALT activity (Fig. 5B), associated with a lower liver damage and fibrosis than the one observed in control (PBS) infected-mice (Fig. 5C). Accordingly, the decrease in clinical signs was associated with a reduction, from 1 day post-infection (dpi), in the frequency and number of MGL2-expressing F4/80^+^ cells (Figs. 5D,E).

### MGL2^+^ cell depletion abrogates splenic Treg increase induced by the infection

**Figure 6:**
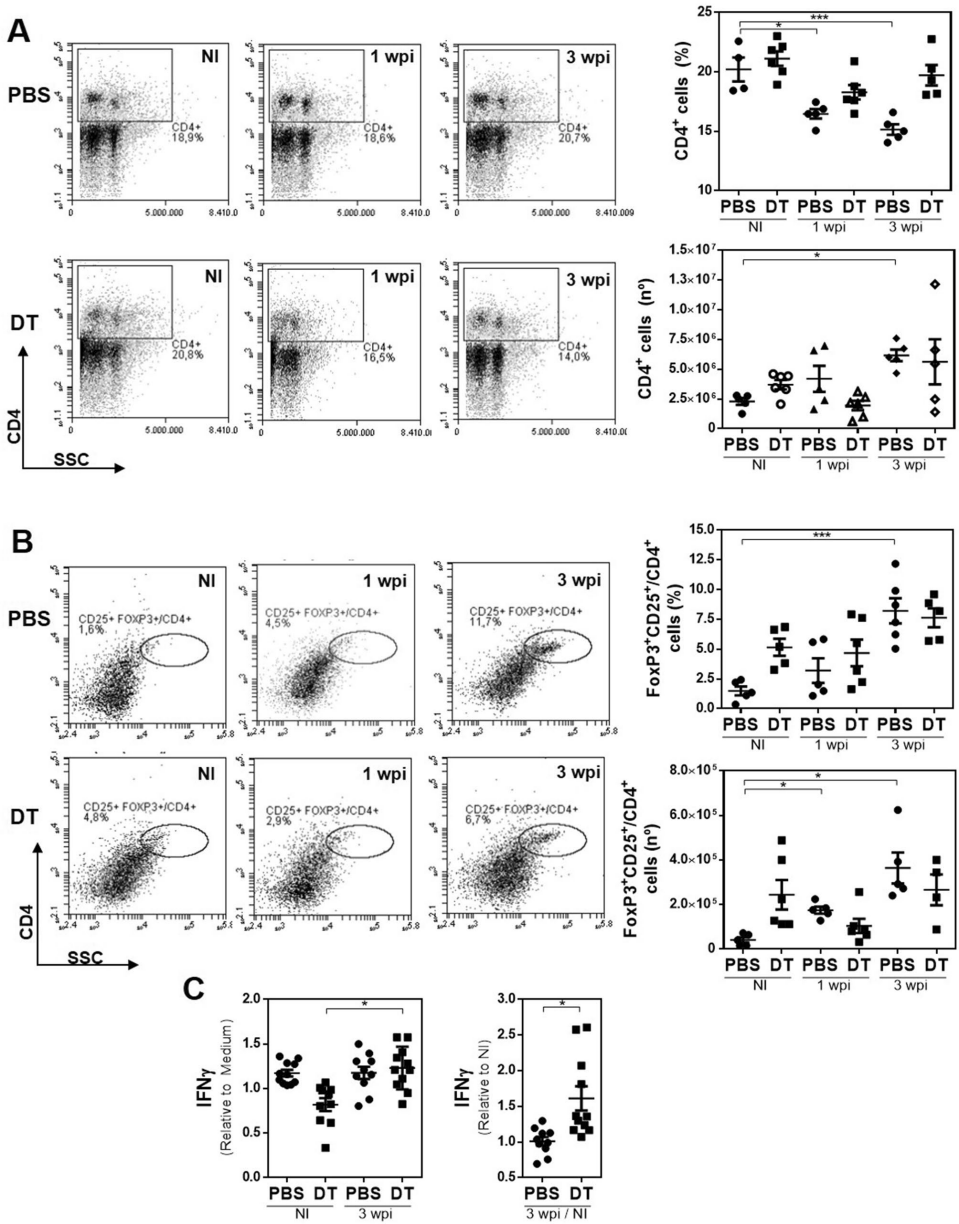
Depletion of F4/80^+^ MGL2^+^ cells during *F. hepatica* infection abrogates splenic Treg expansion. Frequency and number of CD4^+^ cells (**A**) and FoxP3^+ ^CD25^+^/CD4^+^ cells. (**B**) in spleens were determined by flow cytometry. MGL2-DTR mice were infected with 10 *F. hepatica* metacercariae and sacrificed at 1 dpi, 1 or 3 wpi. MGL2^+^ cells were depleted with DT-treatment. Control mice were injected with PBS. Non infected mice were used as controls (NI). (**C**) Production of IFNγ evaluated by specific ELISA on supernatants of splenocyte culture stimulated with PMA/Ionomicin. A representative figure of three independent experiments is shown (± SEM, indicated by error bars). Asterisks indicate statistically significant differences (*p < 0.05, ***p < 0.001).

The depletion of MGL2^+^ cells in MGL2-DTR infected mice prevented the decrease of CD4^+^ T cell percentage in the spleen from 1 wpi (Fig. 6A and Supplementary Fig. 3). In contrast, no significant increase in the CD4^+^ T cell counts was detected in comparison with DT-non treated infected control mice (Fig. 6A). Moreover, the depletion of MGL2^+^ cells in infected mice abrogated the increase in frequency and number of CD4^+^/FoxP3^+ ^CD25^+^ cells (Fig. 6B), suggesting that MGL2^+^ F4/80^+^ cells are critical players in the induction of an adaptive regulatory immune response.

Of note, when splenocytes from infected mice depleted of MGL2^+^ cells were restimulated with molecules derived from the parasite (FhTE), they produced higher levels of IFNγ than those from infected non-depleted mice (Fig. 6C). However, no differences were observed in IL-4 and IL-10 cytokine secretion between infected groups (not shown). We were also unable to detect a difference in IL-4, IFNγ and IL-10 secretion by splenic CD4^+^ T cells between infected mice with or without MGL2^+^ cell depletion by flow cytometry (Supplementary Fig. 3).

### Peritoneal MGL2^+^ F4/80^+^APCs from infected mice are essential for the induction of splenic Tregs

**Figure 7:**
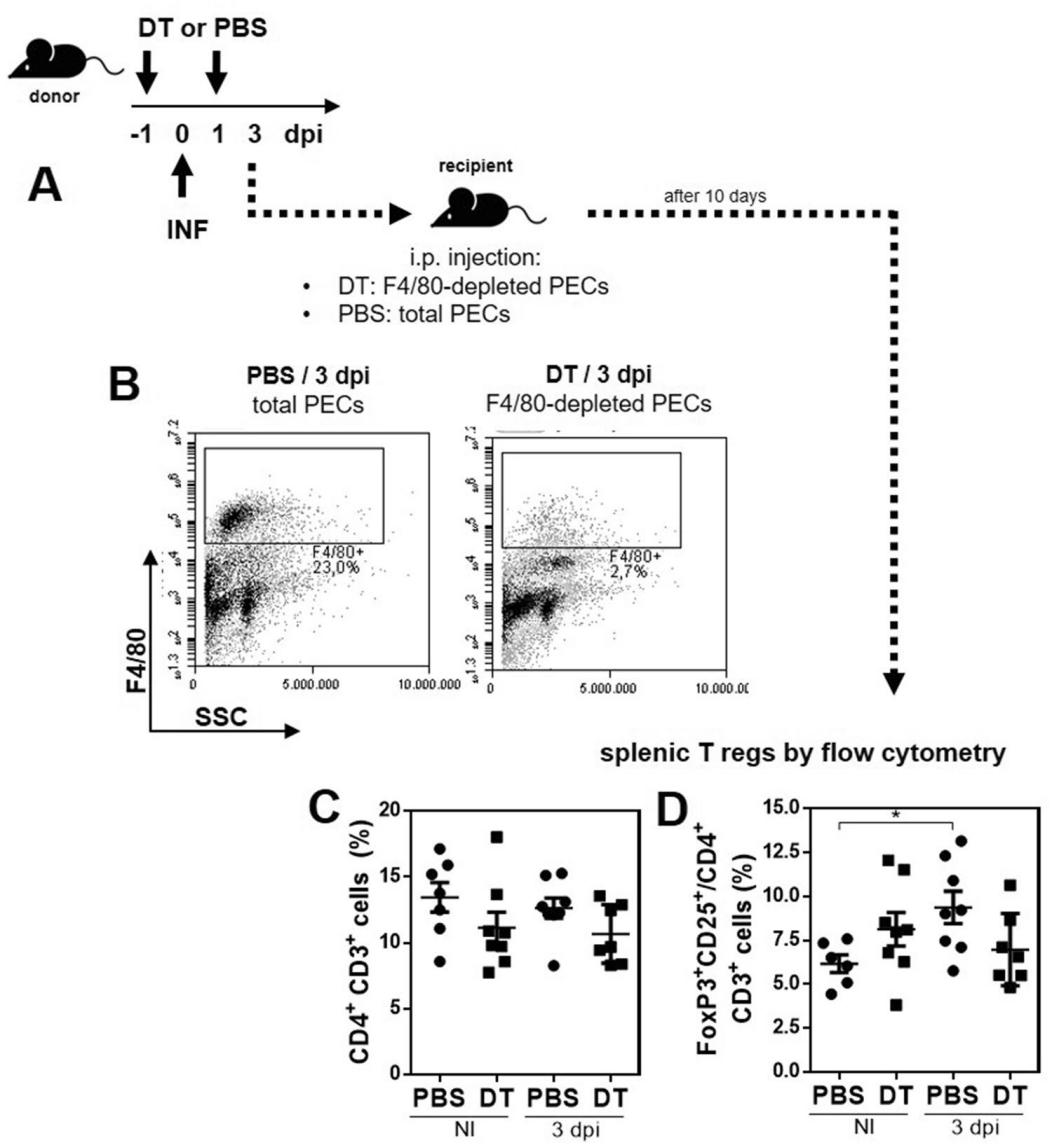
MGL2^+^ F4/80^+^ peritoneal cells induced by *F. hepatica* infection are necessary for Treg induction. (**A**) Adoptive transfer from infected MGL2-DTR mice to recipient non-infected mice. (**B**) F4/80^+^ peritoneal cell depletion with DT treatment after 3 dpi. Frequency and number of CD4^+^ CD3^+^ cells (**C**) and FoxP3^+ ^CD25^+^/CD4^+^ (**D**) cells in spleens from recipient mice determined by flow cytometry. A representative figure of two independent experiments is shown (± SEM, indicated by error bars). Asterisks indicate statistically significant differences (*p < 0.05).

To confirm the role of MGL2^+^ F4/80^+^ peritoneal APCs induced by *F. hepatica* infection in the generation of Tregs in the spleen, we infected MGL2-DTR mice and treated them either with DT to deplete MGL2^+^ cells or with PBS as control. After 3 dpi, peritoneal cells from both groups of mice were adoptively transferred to the recipient non-infected mice (Fig. 7A,B). The presence of CD4^+^ CD3^+^ T cells (Fig. 7C) and Foxp3^+^ CD24^+^/CD4^+^ T cells (Fig. 7D) in the spleen of recipient mice was analyzed by flow cytometry revealing that the group transferred with F4/80^+^ cells presented an increase in splenic Tregs, while depletion of F4/80^+^ cells in infected mice did not induce and increase in splenic Tregs.

## Discussion

Various studies have independently demonstrated the role of CLRs in recognizing, internalizing and signaling upon the stimulation with helminth glycoconjugates^18,26^. The uptake and the internalization of parasite molecules are crucial to allow the antigen processing and presentation that may influence the immune response and promote parasite survival in the host^20,27,28^. In this work, we focused on the role of peritoneal MGL2^+^ myeloid APC during *F. hepatica* infection in inducing splenic Tregs in vivo. Macrophages and DCs represent heterogeneous myeloid cell populations specialized in antigen presentation. However, DCs are unique in their capacity to orchestrate the adaptive immune response by activating naïve T cells and inducing their differentiation into different effector T cells depending on the pathogen^29,30^. Both macrophages and DCs can secrete pro-inflammatory or anti-inflammatory cytokines since they exhibit functional plasticity that enables them to adapt to various local conditions and to restore homeostasis after inflammation^29–31^. Here, we show that MGL2^+^ F4/80^+^ cells are recruited to the peritoneal cavity likely through an increase in CCR2, the receptor of the monocyte chemoattractant proteins 1 and 3^32,33^. Indeed, CCR2 is a chemokine receptor associated with the recruitment of inflammatory monocytes in parasite infections^34,35^ and it mediates the cell migration from the bone marrow and the recruitment of monocytes, myeloid suppressor cells^32,33^ or monocyte-derived DC-like cells^36^ that express MHC II together with CD11c and F4/80 into damaged tissue or inflamed tissues.

MGL is a type II transmembrane protein expressed on professional APC. Even though there is only one MGL in humans (hMGL), two orthologues are present in mice that possess different glycan specificity (mMGL1 and mMGL2)^37,38^. Both mMGL2 and hMGL display similar specificity for terminal GalNAc moieties, including the Tn antigen (αGalNAc-O-Ser/Thr) and LacDiNAc (GalNAcβ1-4GlcNAc)^39^ and can recognize glycoconjugates from helminth parasites, such as *Schistosoma mansoni*^40^, *Trichuris suis*^41^ and *Taenia crassiceps*^42^. Furthermore, it has been proposed that MGL2^+^ dermal DCs are specialized in inducing Th2 responses both in allergy and helminth-infection models^42^.

In order to confirm that MGL2^+^ cells that express both CD11c and F4/80 may constitute a population of DCs, we evaluated the expression of MerTK, a macrophage specific molecule^43^. Due to the low expression of MerTK and high levels of Sirpα, PD-L1, IL-10 and TNFα, we suggest that the CD11c^+^ F4/80^+^ CCR2^+^ cells, that also express MGL2 during *F. hepatica* infection, constitute a population of monocyte-derived DCs with regulatory properties. Moreover, as previously described by our team, and in accordance with our studies performed in MGL2-DTR transgenic mice, regulatory DCs from *F. hepatica* express high levels of Sirpα and IRF4^6^. Both IRF4 and Sirpα participate in immunoregulation and can promote Treg differentiation^44–46^. In *F. hepatica* infection in mice, MGL2^+^ regulatory DCs can suppress Th1 differentiation and induce the production of IL-10 by CD4^+^ T cell lymphocytes in vitro^6^. These regulatory functions could be mediated by PD-L1, the ligand of PD-1, expressed on regulatory DCs. This molecule, together with PD-L2, is an immune inhibitor receptor expressed on T cells that limits cell proliferation, induces Treg differentiation and serves to maintain immune homeostasis^47,48^. Indeed, PD-L2 negatively regulates Th1-mediated immunopathology during *F. hepatica* infection^18^. Therefore, PD-L1 on DCs could play a role in controlling the induction of parasite-specific immunity that allows its survival. In addition, a previous work demonstrated that CCR2^+^ cells recruited in the early stage of infection with *T. crssiceps* express PDL-1, and suppress T cell proliferation in vitro^49^. Furthermore, molecules from the tapeworm *Hymenolepis diminuta* induce a CCR2-dependent recruitment of myeloid monocyte-like cells that express high levels of PD-L1 to the peritoneum. These cells enhance both IL-10 and IL-4 secretion by activating T cells in vitro and have an immunosuppressive role in vivo^50^.

In a recent report we have shown that F4/80^+^ myeloid cells in the peritoneum of infected animals express HO-1^51^, the inducible rate-limiting enzyme involved in the catabolism of free heme^52^ that promotes anti-inflammatory cytokine secretion^53,54^. In fact, HO-1 expression by DCs induces the production of IL-10 and inhibits T cell proliferation^55^. In *F. hepatica* infection it induces production of IL-10 which is necessary for parasite establishment in the host^51^. Of note, hepatocyte growth factor receptor induces Ras-dependent upregulation of both HO-1 and PD-L1 in cancer^56,57^. In addition, myeloid cells expressing both HO-1 and PD-L1 in breast tumors suppress T cell activity^58^. However, further experiments are necessary to explore whether MGL2 signaling is related with HO-1 or PD-L1 expression in peritoneal DC-like cells in *F. hepatica* infection in mice.

We have previously demonstrated that MGL2^+^ cells induce the production of IL-10 by CD4^+^ T cells^6^. To explore the involvement of these cells in the differentiation of Tregs, we depleted MGL2^+^ cells from *F. hepatica* infected mice. We found that they are crucial for infection and differentiation of Tregs, since an increased number of CD4^+^/FoxP3^+^CD25^+^ cells was found in the spleens of infected mice and their depletion abrogated the Treg expansion induced by the infection. Furthermore, adoptive transfer of peritoneal cells from infected mice with or without MGL2^+^ cell depletion revealed that MGL2^+^ DCs from infected mice can acquire an immunoregulatory program that licenses them to induce Treg differentiation. Nevertheless, the parasite molecules that trigger this immunoregulatory pathway on peritoneal MGL2^+^ myeloid cells during *F. hepatica* infection have not yet been identified. Indeed, although the identification of differentially-expressed genes involved in the glycosylation process of *F. hepatica* proteins has been performed^59^ the immunomodulatory role of glycans in certain proteins is still unknown. On the contrary, several studies have identified the glycan motifs from *F. hepatica* that can interact with CLRs on these cells, such as the Tn antigen that interacts with MGL2^6,10^. Other glycans such as mannosylated glycoconjugates favor anergic T cells or Tregs that silence the immune system of the host through CD209 (or DC-SIGN)^10^. In addition, MR was described to interact with *F. hepatica* molecules and to mediate the partial inhibition of TLR-induced maturation of bone marrow-derived DCs^13,19^. Last, both MR and Dectin-1 immunomodulate Arginase-1 and PD-L2 expression and TGFβ production by macrophages in response to *F. hepatica* excretory–secretory products^12,18^. These data suggest that the parasite targets more than one CLR in order to evade immunity. Further experiments are needed to determine whether different factors participate in this process, such as glycans in parasite microvesicles^60,61^. In addition, other works have reported the anti-inflammatory role of *F. hepatica* molecules in training the innate immunity of macrophages^62^. However, the role of glycans in the induction of epigenetic imprinting of cells has not yet been determined**.**

In conclusion, we demonstrate that MGL2^+^ DC-like cells are recruited to the peritoneum during experimental *F. hepatica* infection in mice. They express immunoregulatory molecules that associate with an increase in clinical signs and expansion of Tregs, thus, favoring infection. Altogether, these results suggest that strategies based on MGL2 targeting could be helpful in the control of fasciolosis.

## Methods

### Ethics statement

Adult worms were collected during the routine work of a local abattoir (Frigorífico Carrasco) in Montevideo (Uruguay)*.* Protocols were approved by the Uruguayan Committee on Animal Research (Comisión Nacional de Experimentación Animal, CNEA, Uruguay).

### Mice

MGL2-DTR six- to eight-week-old mice were purchased from Jackson Laboratory (USA). They were used to analyze the role of MGL2^+^ cells during *F. hepatica* infection. MGL2^+^ cells can be depleted with DT injection^24^. Animals were kept in the animal house (URBE, School of Medicine, UdelaR, Uruguay) with water and food supplied ad libitum. Mouse handling, care and experiments were carried out in compliance with institutional guidelines and regulations from the National Committee on Animal Research (CNEA, http://www.cnea.org.uy/, National Law 18.611, Uruguay) and in accordance with ARRIVE guidelines. Procedures involving animals were approved by the Universidad de la República’s Committee on Animal Research (Comisión Honoraria de Experimentación Animal, CHEA Protocol Number 070153–000,811-19).

### Parasite infection, animal treatment and sample obtention

Ten *F. hepatica* metacercariae (Montevideo, Uruguay) were orally administered per mouse. Viability of metacercariae was analyzed by in vitro excystment (> 70%) as previously evaluated^63^. Mice were bled at 3 wpi and peritoneal exudate cells (PECs), spleens, and livers were removed after either 1 dpi, 1 or 3 wpi, depending on the experiment. Non-infected animals were used as controls (0 dpi). To evaluate parasite infection while depleting MGL2^+^ cells, metacercariae were administered on day 0 into MGL2-DTR mice that were previously intraperitoneally (i.p.) injected with diphtheria toxin (DT, 0.5 µg/mouse) (The Native Antigen Company, USA) or PBS on day − 1 and every 2 or 3 days until the end of the experiment. Each experimental group contained at least six mice. PECs and spleens were processed as already described^64^. Red cells were lysed with ammonium chloride potassium buffer. The alanine aminotransferase (ALT) activity in sera was used to quantify liver damage and was determined with a colorimetric commercial kit (Spinreact, Spain) according to the manufacturer's instructions. The infection severity was assessed with a defined clinical score according to the following parameters: presence or absence of peritoneal hemorrhage, presence of macroscopic liver damage and splenomegaly, and the amount of cell content in the peritoneal cavity^51,64,65^, where the minimum score was 0 and the maximum was 10 (described in detail in Supplementary Table 1).

### Flow cytometry

Cell suspensions from PECs and spleens were washed twice with PBS containing 2% FBS and 0.1% sodium azide (FACS buffer) and stained with specific antibodies for 30 min at 4 °C as previously published^51^. The following antibodies (Biolegend, USA) were used: anti-Sirp⍺ (P-84), -CD11c (N418), -CCR2 (SA203G11), -PD-L1 (10F9G2) and ICOS-L (HK5.3). Expression of FoxP3, HO-1 and IL-10 was analyzed by intracellular staining by permeabilizing with Cytofix and Perm wash buffers (Biolegend, USA), incubated with anti-IL-10 (JES5-1E3), -FoxP3 (MF14), TNFα (MP6-XT22) and HO-1 (clone ab13248 from Abcam, USA) specific antibodies. Analyses were performed using a BD Accuri C6 Plus cytometer and software (BD-Biosciences, USA).

### Proliferation assay and cell culture

Parasite protein extract (FhTE) was prepared from live adult flukes obtained from infected bovines as previously published^51^. Splenocytes (0.5 × 10^6^/well) from infected mice or uninfected naïve mice (control group) were cultured for 5 days at 37 °C and 5% CO_2_, in RPMI-1640 with 400 µg/ml glutamine (Capricorn Scientific, Germany) complete medium containing 10% heat-inactivated fetal bovine serum (FBS, Capricorn Scientific, Germany), 50 mM 2-mercaptoethanol, 100 U/ml penicillin, 0.1 mg/ml streptomycin (Merk, Sigma-Aldrich, USA) in presence or absence of FhTE (75 µg/ml) as previously described^9^. An IFNγ-specific sandwich ELISA assay (Biolegend, USA) was used to quantify IFNγ levels in culture supernatants.

### Adoptive transfer of peritoneal cells from infected mice

To evaluate the capacity of MGL2^+^ cells to induce Tregs, 6 MGL2-DTR mice that were i.p. injected with DT (0.5 µg/mouse) or PBS on days − 1, + 1 and + 3, were infected with 10 *F. hepatica* metacercariae on day 0. As control groups non-infected MGL2-DTR mice injected either with DT or PBS were used (n = 6). At 4 dpi, PECs from the four groups were collected, red cells were lysed and remaining cells were counted. Depletion of peritoneal MGL2^+^ cells with DT treatment was verified by flow cytometry. 1 × 10^6^ cells were i.p. injected in recipient non-infected MGL2-DTR mice that did not receive DT treatment. After 10 days, spleens were removed and CD3^+^ CD4^+^/FoxP3^+^ CD25^+^ cells were analyzed by flow cytometry as described above.

### Statistical analysis

The obtained results were expressed as mean ± SEM. Statistical analyses were performed with GraphPad Prism version 6.04 for Windows (GraphPad Software, USA) was used to perform statistical analyses. Results were analyzed using one-way ANOVA followed by Tukey’s test, or two-tailed student's *t*-test, depending on the experiment. Significant differences shown by asterisks were considered when *p < 0.05, ** *p* < 0.01, *** *p* < 0.001, **** *p* < 0.0001.

## Supplementary Information

Supplementary Information.

## Data Availability

All data generated or analyzed during this study are included in this published article or available upon request (and its Supplementary Information files).
