# Supplementary material for: Macrophage Gal/GalNAc lectin 2 (MGL2)+ peritoneal antigen presenting cells during Fasciola hepatica infection are essential for regulatory T cell induction
Source: Sci Rep. 2022 Oct 21;12:17661. doi: 10.1038/s41598-022-21520-w (PMC9587262; doi:10.1038/s41598-022-21520-w)
Supplement: Supplementary file 1 — Supplementary Information. [file 41598_2022_21520_MOESM1_ESM.pdf]

Supplementary Table 1. Clinical score of *F. hepatica* infected mice used in this study.

Ascites		Spleen		Number of lesions / hepatic lobe		Liver lobes	
**Score**	**Description**	**Score**	**Size**	**Score**	**Description**	**Score**	**Description**
0	None (normal cell content)	0	Normal	0	None	0	Healthy
1	Mild (medium cell content)	1	Splenomegaly (< x2)	1	< 3 lesions	1	1 hepatic lobe affected
2	Moderate (high cell content)	2	Splenomegaly (> x2)	2	> 3 lesions	2	> 2 hepatic lobes affected
3	Severe (high cell and blood content)			3	Complete affection of lobes		

Maximal score is 10

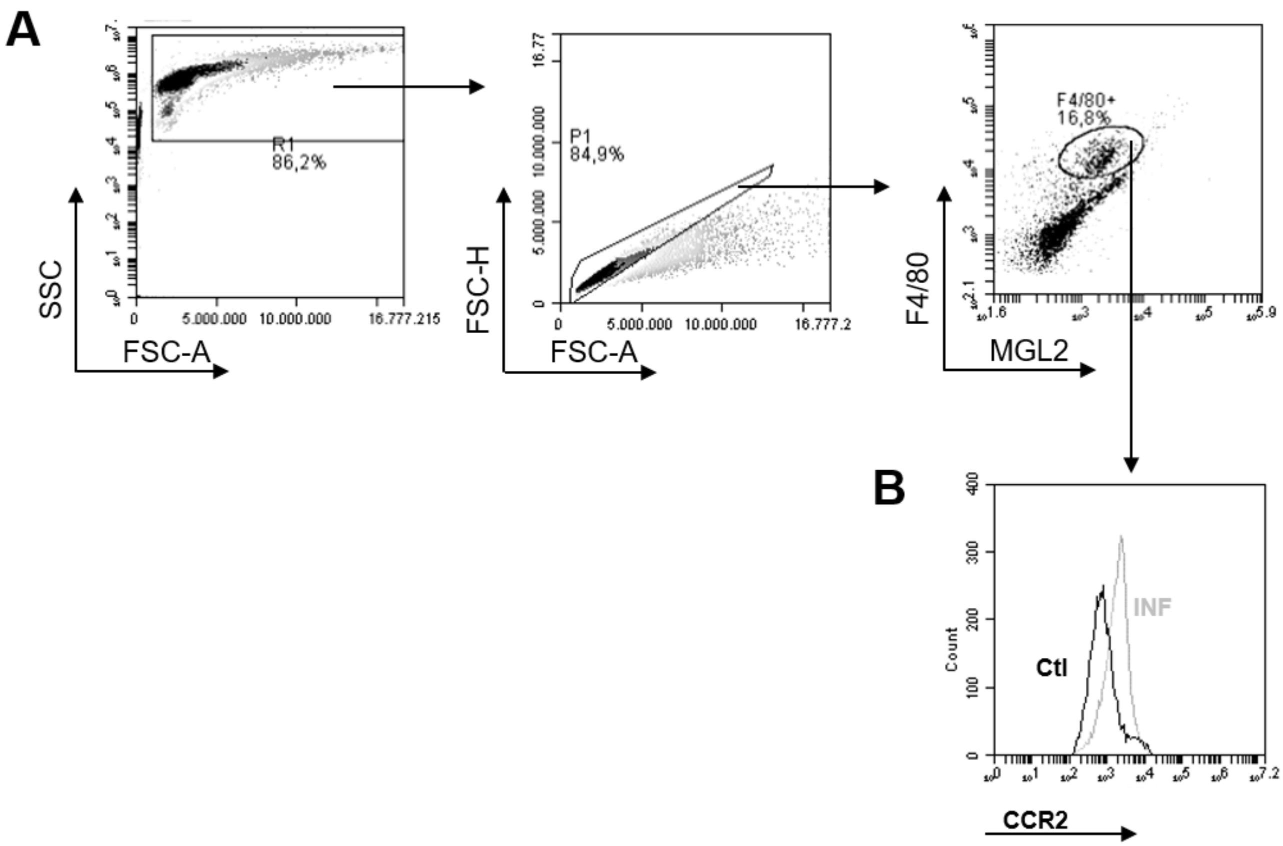

Supplementary Figure 1. Gates used for flow cytometry analyses of PECs from infected and non-infected mice. PEC suspensions were gated according to side and forward scatter and singlets were selected. Cells were stained with anti-F4/80 and -MGL2 antibodies **(A).** CCR2 expression (shown as an example) was determined according to Median Fluorescence intensity on F4/80^{+} cells after staining with a specific antibody in non-infected (Ctl) and infected mice (INF) **(B)**.

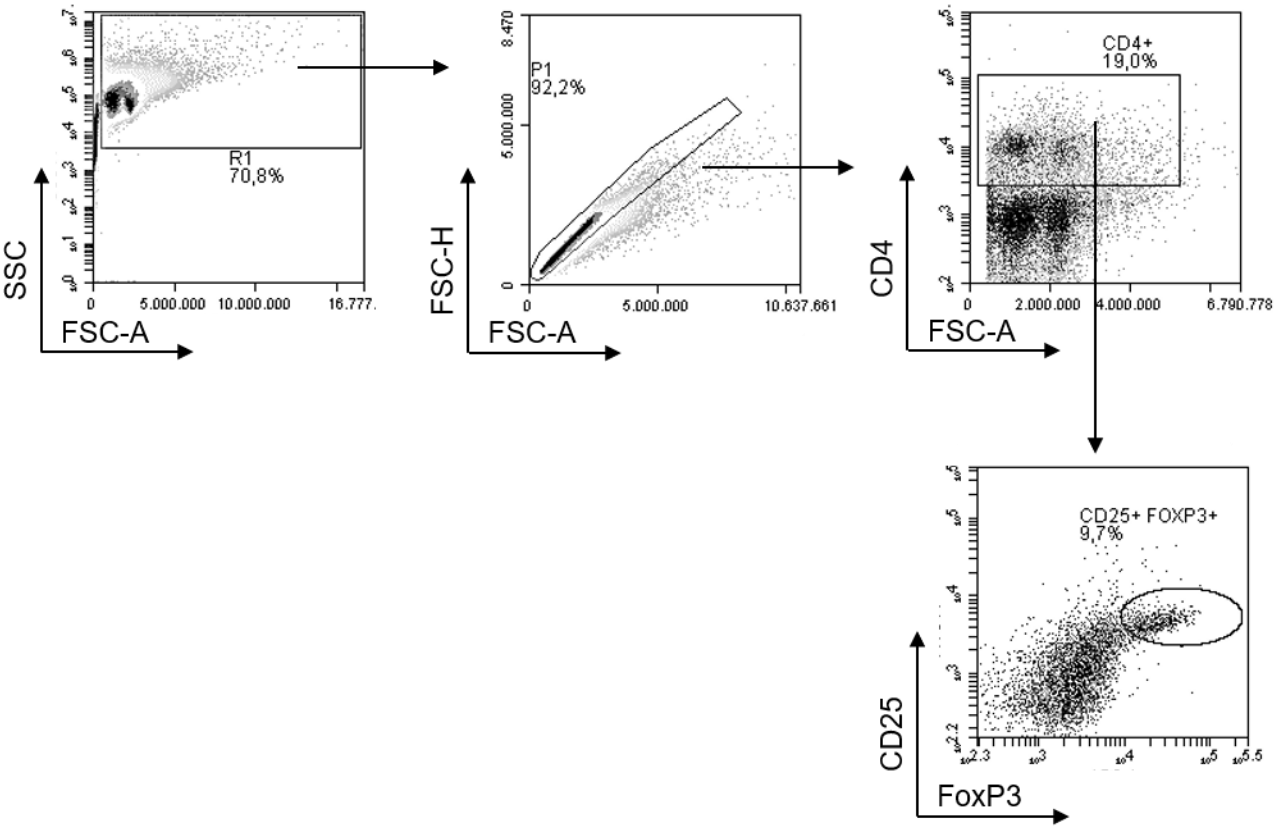

Supplementary Figure 2. Gates used for flow cytometry analyses of spleens from infected and non-infected mice. Splenocyte suspensions were gated according to side and forward scatter and singlets were selected . Cells were stained with anti-CD4, -CD25 and -FoxP3 antibodies.

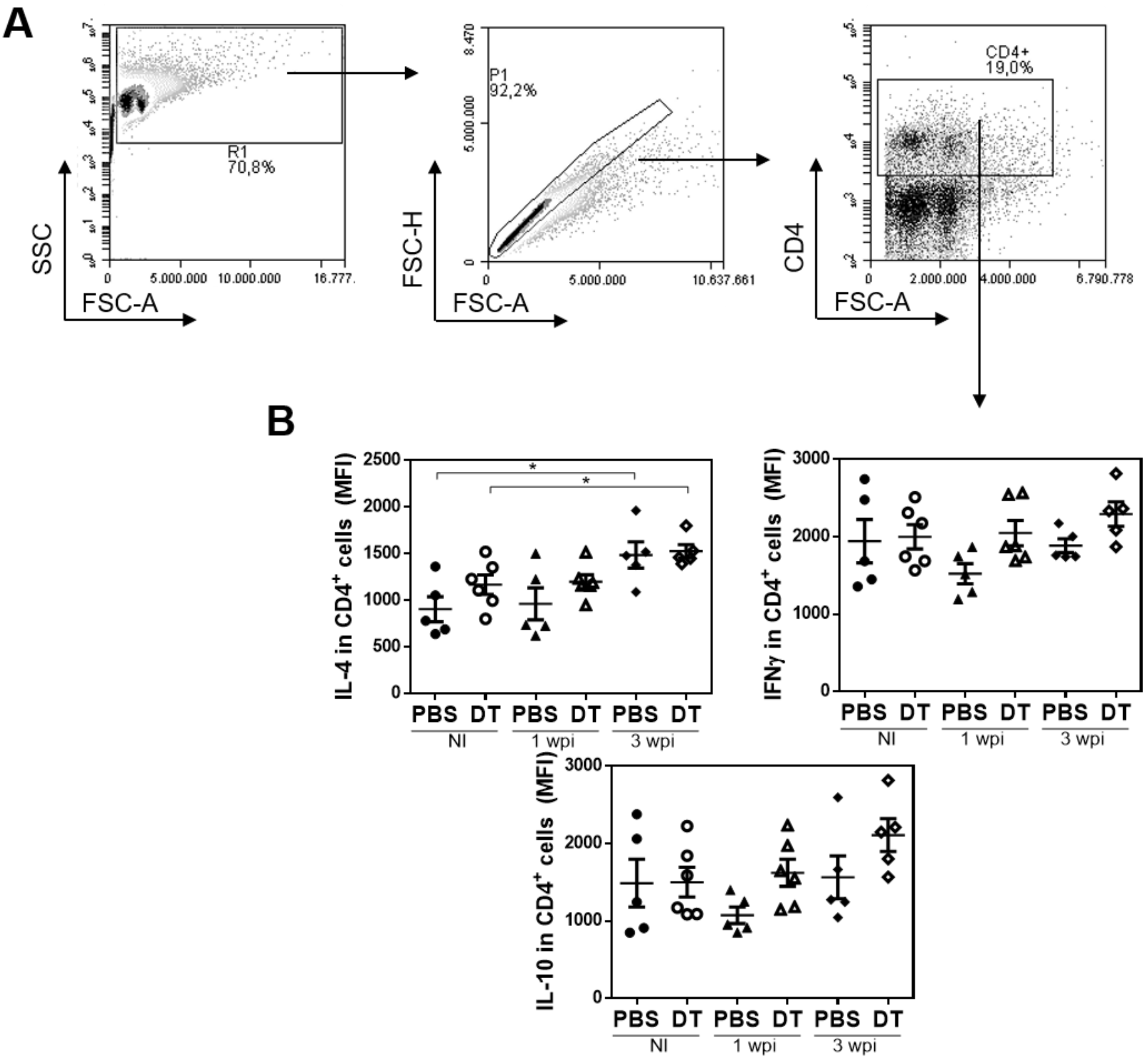

Supplementary Figure 3. Cytokine expression in $CD4^+$ splenocytes from infected and non-infected mice. Splenocyte suspensions were gated according to side and forward scatter and singlets were selected. Cells were stained with anti-CD4, -IL-4, -IFNγ and -IL-10 antibodies **(A)**. Cytokine expression was represented as Median Fluorescence Intensity (MFI) in $CD4^+$ cells from infected and non-infected mice in the presence or absence of DT-treatment to deplete $MGL2^+$ cells **(B)**.
